# Optimization and Planning of Emergency Evacuation Routes Considering Traffic Control

**DOI:** 10.1155/2014/164031

**Published:** 2014-05-29

**Authors:** Guo Li, Lijun Zhang, Zhaohua Wang

**Affiliations:** ^1^School of Management and Economics, Beijing Institute of Technology, Beijing 100081, China; ^2^Center for Energy and Environmental Policy Research, Beijing Institute of Technology, Beijing 100081, China

## Abstract

Emergencies, especially major ones, happen fast, randomly, as well as unpredictably, and generally will bring great harm to people's life and the economy. Therefore, governments and lots of professionals devote themselves to taking effective measures and providing optimal evacuation plans. This paper establishes two different emergency evacuation models on the basis of the maximum flow model (MFM) and the minimum-cost maximum flow model (MC-MFM), and proposes corresponding algorithms for the evacuation from one source node to one designated destination (one-to-one evacuation). Ulteriorly, we extend our evaluation model from one source node to many designated destinations (one-to-many evacuation). At last, we make case analysis of evacuation optimization and planning in Beijing, and obtain the desired evacuation routes and effective traffic control measures from the perspective of sufficiency and practicability. Both analytical and numerical results support that our models are feasible and practical.

## 1. Introduction

Major emergencies, concerning accidents such as natural disasters, public health events, and social abrupt affairs, occur in a very short period of time and always cause serious damage to the society. They are all characterized by suddenness, uncertainty, and serious harmfulness, and have been a big challenge for sustainable development of the human society.

In recent years, various disasters occur frequently. Therefore, countries all over the world attach great importance to emergency management. China has entered into the high-incidence season of emergencies and will face the baptism brought by emergencies in a long period of time [1]. With the further acceleration of urbanization, the population is more concentrated, so how to respond effectively and timely to emergencies is especially important. In most emergency management, how to evacuate people to safety zone becomes a crucial step. For example, cyclohexane spill that occurred in 1976 in Seveso, Italy, rained a cloud of dioxin on surrounding communities, and the local authority organized a evacuation of 220,000 people. Similarly, when liquid chlorine cylinder explosion accident occurred in southern China in 1979, emergency evacuation was also implemented to move 60,000 people away. Likewise, in 1984, after Mexico City gas storage tank exploded, 350,000 people were evacuated [2].

The evacuation issues can be divided into two different types: small scale evacuation and long-distance regional evacuation [3]. Small scale evacuation generally refers to a kind of evacuation when emergencies affect small area swiftly and violently, such as explosion within a finite range, house collapsing, fire breaking out in shopping malls, and so on. Generally, evacuation of this kind mainly deals with evacuation on foot, rather than using vehicles. In contrast, long-distance regional evacuation refers to a form of evacuation implemented along with the appearance of a wide range of emergencies, such as leakage and diffusion of harmful gases and earthquakes. Since the evacuation route is long-distance, this kind of evacuation generally needs vehicles for transport. In addition, according to the difference of subject and actions taken after emergencies happen, the long-distance regional evacuation can be divided into autonomous evacuation, recommended evacuation, and mandatory evacuation [4]. Autonomous evacuation and recommended evacuation are carried out spontaneously and preparedly by people when they get messages or notifications of disasters, in which case the alert time is comparatively longer, while mandatory evacuation responds to emergencies having the need of urgent evacuation and generally commanded by governments or related departments. All of the evacuations considered in this paper belong to the latter situation.

The rest of our paper is organized as follows.  Section 2 presents a review of related literature.  Section 3 deals with one-to-one evacuation optimization problem based on MFM.  Section 4 develops one-to-one evacuation model based on MC-MFM.  Section 5 explores the one-to-many evacuation problem. Case analysis is reported in Section 6, and finally some concluding remarks are given in Section 7.

## 2. Literature Review

Generally, scholars mainly tend to consider long-distance regional evacuation or mandatory evacuation first, figuring evacuation routes as network diagrams to achieve shortest routes, maximum flow, or minimum-cost maximum flow, such as adopting contraflow transportation network reconfiguration [5], establishing the cell-based evaluation network model [6], analyzing multifactors with the construction of a system which involves the evacuation network [7], building the network flow model for lane-based evacuation routing [8], and solving evacuation problems by using dynamic network flows [9, 10]. Lots of researchers have not only optimized evacuation route, but also explored many methods. The representatives refer to Choi et al. [11], Han [12], Hoppe and Tardos [13], Klüpfel et al. [14], Theodoulou [15], and Adewmi and Garba [16]. With regard to multiobjective evacuation routing, Stepanov and Smith [17] present a methodology for designing optimal routing policies for emergency evacuation planning (EEP) through building an integer program (IP) model. On the problem of roadway deployment, Pal and Bose [18] propose an integer model to find the best location and assign response vehicles to those depots under reliability constraints. Saeed and Ram [19] examine the evacuation routing problems by proposing a two-step approach that consists of an incapacitated integer multi-commodity network model and a computational algorithm. Considering nonlinear relations, Zhang et al. [20] explore a min-cost-pursued swapping dynamic (NMSD) system to model the evolution of selfish routing games on the traffic network.

Besides, many topics related to emergency evacuation also have been discussed. Regarding the evacuation problem in surrounding areas of nuclear power plant, Dunn and Newton [21] investigate how to evacuate people as far as possible within the prescribed time, looking upon the evacuation problem as a maximum flow problem to find the optimal evacuation route. As to the evacuation in an earthquake, Yamada [22] describes route optimization of neighborhoods as the shortest route problem and the minimum cost flow problem, respectively. Campos and da Silva [23] try to reduce the conflicts between finding the evacuation flows and increasing the traffic capacity of evacuation route. They treat the ratio of traffic capacity and travel time as a measurable index of traffic performance. On the issue of hurricane evacuation, Dixit and Radwan [24] provide a new and innovative technique with a “network breathing strategy” at destinations after dictating when to schedule evacuation orders and capacities required on different routes. Based on the data of earthquakes in 1994, Northridge and 1995, Kobe, Koike et al. [25] provide a probabilistic approach to simulate the evacuation scenario along the streets crowded with evacuation people. Specifically, the ratio of fire-proof structures, ignition rates from fire gutted houses, and population density are all assumed in a probabilistic manner. In view of blocking effects on crowd movement, Luh et al. [26] establish a new macroscopic network-flow model assuming that fire, smoke, and psychological factors can evoke crowd's desire to escape at the expected flow rate and then develop a divide-and-conquer approach to reduce computational complexity and reflect psychological changes.

As for effective solutions for route optimization, Hamacher and Tjandra [27] all systematically summarize the dynamic network flow models, which are widely used in the optimization of human organization evacuation planning, including the dynamic maximum flow model, the fastest dynamic flow model, and the global maximum dynamic flow model. Besides, they analyze the utilization potentiality of these models in regional and extensive evacuation. Mamada et al. (2003) [28] treat the optimization of evacuation routes and the assignment of departure times as the fastest flow problem. With the assumption that the traffic starting from the source node could only depart for a designated destination, they provide a algorithm to find the fastest flow in the tree network diagram. Similarly, Lu et al. [29] regard the evacuation planning that includes departure time, evacuation routes, and ending point selection as the fastest transshipment problem, and propose a heuristic algorithm on the basis of network figural representation to find the optimal organization planning directly. Comprehensively, Lin [30] constructs a framework integrated with optimizations, evaluations, and adjustments, aiming to achieve the optimization of evacuation organization planning. Fuellerer et al. [31] consider that, on two-dimensional level, successive routing of the vehicles in the road network by a colony algorithm can satisfy customers' demands with the consideration of the freight loading factor. Likewise, Leung et al. [32] develop an extended guided tabu search (EGTS) method and a new heuristic packing algorithm for the two-dimensional loading vehicle routing problem, which can help tabu search to escape from local optimum effectively. From the perspective of information quality and evolution, Pillac et al. [33] present a general description of dynamic routing and introduce the notion of degree of dynamism, and then bring out a comprehensive review of applications and solution methods for dynamic vehicle routing problems. When capacity constraints exist, Xu et al. [34] propose a stochastic user equilibrium assignment model for a schedule-based transit network, through which they can simultaneously predict how passengers choose their transit vehicles to minimize their travel costs and estimate the associated costs. And they also find that when a connection segment reaches its capacity level, the Lagrange multipliers of the mathematical problem give the equilibrium passenger overload delays in this transit network.

In addition, some scholars formulate models to find the concrete flow and path from source nodes to designated destinations. To minimize the end-to-end delays for the specific routing mechanism, Grimmell and Rao [35] discuss the problem that deals with the transmission of a message from a source node to a designated destination over a network with propagation delays and dynamic bandwidth constraints on the links. They make available bandwidth for each link specified as a piecewise constant function and present for each message forwarding. In order to identify the network paths accurately, Murtaza et al. [36] present a new mechanism for detecting shared bottlenecks between end-to-end paths in a network, which is based on the well-known linear algebraic approach-singular value decomposition (SVD). Specific to the evacuation in the feeder-bus network, Deng et al. [37] extend the demand pattern M-to-1 (i.e., multiple bus stops and a single station) to M-to-M, considering the passenger travel cost. Moreover, they present a new genetic algorithm to determine the optimal feeder-bus operating frequencies under strict constraints, and finally find that demand distributions also should be considered when designing a feeder-bus network.

The literature mentioned above mostly converts evacuation problems to dynamic or static network model by finding shortest routes, maximum flow, and minimum-cost maximum flow. However, intuitive calculation makes the entire emergency management on evacuation too rigid, and there are many actual-world factors that cannot be fully reflected in the model.

First of all, intersections are everywhere in reality, so the impacts of them should be considered during evacuation process in avoidance of possible time delays. Although it may make computing process more complex, it is better than ignoring intersections' impact. By the way, the complexity of intersections does not only reflect on the limitation of certain straight road sections, but also on that of different turning routes. Being different from classic emergency evacuation management, this paper fully examines the influence of intersections.

Secondly, previous researches on impacts of traffic management and control measures are very few. The actual evacuation may be affected by many aspects of constraints, which are all objective conditions that constrain the evacuation carried on. Therefore, control measures should be considered in the model, such as duplicated row, single row, and forbidding for passing as well as some uncertain real-time management and control measures, like signal control and real-time directing.

Finally, regarding the problem of establishing evacuation models, two different kinds of evacuation, namely, evacuations from one source node to one designated destination(one-to-one evacuation) and one source node to multiple designated destinations (one-to-many evacuation), also are taken into account according to the actual situation.

## 3. Evacuation Optimization Model Based on MFM

When dangerous situations are unknown to people, the maximum number of evacuations can only be implemented within the capacity limitations, and each batch of evacuation must be the maximum flow. In addition, in order to facilitate the description and calculation, the maximum flow below generally refers to the flow.

### 3.1. Assumptions and Prerequisites

In the network diagram model with the given time and the goal of maximum flow, its basic assumptions and prerequisites are as follows.

1. There is only one source node and one designated destination, and vehicle number in the source node are known.
2. Allowed evacuation time can be obtained by forecasts, and thus evacuation time is given.
3. Traffic capacity of each road section and each intersection's different turnings are known.
4. Limitations of traffic capacity are only valid for one batch, and next batch has new limitations on its traffic capacity.
5. The goal is to maximize evacuation vehicle number under limitations of evacuation time and traffic capacity, namely, to find the maximum flow in a network diagram.

In the above prerequisites, assumption (4) also reflects a characteristic of this modeling. Maximum-flow model generally refers to the model for achieving one-time maximum flow under traffic capacity constraints. Due to the limitations of traffic capacity, maximum flow in multiple times is taken into account in this model. Under this circumstance, batches will be limited, and each batch has the same limitation of traffic capacity. Moreover, the maximum flow includes vehicle number of the *m*th batch and the (*m* − 1)th batch, where *m* = ⌈*T*/*t*⌉.

### 3.2. Notations

Nodes include source nodes, intersections, and evacuation destinations. Arcs represent road sections between the intersections, source nodes, destinations, and adjacent intersections, which convert the entire road network to directive network diagrams *G* = (*V*, *L*, *C*, *U*), where *V* represents a vertex in the figure, expressed by characters *i*, *j*, *k*. The node  1 and node *n* represent the source node and the designated destination, respectively.

 
*L* 
represents the arc, while (*i*, *j*) represents straight line arc from *i* to *j*, and (*i*, *j*, *k*) represents steering arc from (*i*, *j*) to (*j*, *k*) through *j*.
  
*C* stands for the node weight set, and *c*_*ijk*_ represents the traffic capacity of *j* from road section (*i*, *j*) to road section (*j*, *k*) through *j*.
 
*U* is the arc weight set, and *u*_*ij*_ stands for the traffic capacity of road section (*i*, *j*).
 
*m* represents the final evacuation batch.
 
*f* ^0^ is the given flow of source node.
 
*f*_*i*_ is the maximum flow of *i*th batch.
 
*f*_*m*_ is the flow of final batch evacuation.
 
*f*_*T*_ represents the total number of evacuation flow.
 
*f*_*ij*_ represents the flow on arc (*i*, *j*).
 
*f*_*ijk*_ represents the flow from the arc (*i*, *j*) to arc (*j*, *k*) through *j*.

### 3.3. Formulations Based on MFM

Based on the above assumptions and definitions, this evacuation can be expressed as the following models:

(1)  Max⁡ fT=∑i=1mfi=(m−1)fi+fm

(2) $$\begin{array}{c} \;\;\;\;=\left(m-1\right)\overset{n}{\underset{i=1}{\sum }}f_{1i}+f_{m} \end{array}$$

(3) $$\begin{array}{c} \text{s}.\text{t}.\;\;0\leq \;f_{ij}\leq u_{ij\;},\;\left(i,j\right)\in L \end{array}$$

(4) $$\begin{array}{c} 0\leq \;f_{ijk}\leq c_{ijk},\;\left(i,j\right),\left(j,k\right)\in L \end{array}$$

(5) $$\begin{array}{c} \overset{n}{\underset{i=1}{\sum }}f_{1i}=\overset{n}{\underset{i=1}{\sum }}f_{in} \end{array}$$

(6) $$\begin{array}{c} f_{ij}=\overset{n}{\underset{k=1}{\sum }}f_{ijk},\;\left(i,j\right)\in L \end{array}$$

(7) $$\begin{array}{c} f_{jk}=\overset{n}{\underset{i=1}{\sum }}f_{ijk},\;\left(j,k\right)\in L \end{array}$$

(8) $$\begin{array}{c} 0\;\leq f_{m}\leq f_{i} \end{array}$$

(9) $$\begin{array}{c} f_{ij}\geq 0,\;f_{ijk}\geq \;0\;\left(i,j\right),\;\left(j,k\right)\in L. \end{array}$$

While formula (1) is the objective function, formula (2) is its decomposition type; formulas (3) and (4) are constraints of traffic capacity of road sections and intersections, respectively; formula (5) means that the flow of each batch reaching the destination equals the flow departing from the source node; formulas (6) and (7) are the flow conservative constraints of each intersection; formula (8) represents that the flow of the final batch is not more than the maximum evacuation flow of front batches; formula (9) is nonnegative constraints.

### 3.4. Solutions

The model established above is not linear, so linear programming cannot be used directly to find the solution. Certainly, this model can be converted into one or multiple linear programming models, but obviously the solution process will be more complex. Comparatively speaking, it is much more convenient to establish the network diagram model.

From the view of formula 1, the key point of the solution is to obtain *m*, *f*_1_, and *f*_*m*_.

In fact, *f*_1_ can be obtained by the MFM in the network diagram, using labeling method. Traditional solution to find maximum flow in network diagram is to label points in sequence to obtain the augmented chain. However, the solution of this model is to label arcs. Besides, arcs can be labeled including straight line arcs and steering arcs at the intersection on road sections. In this model, let *p* represents the set of the augmented routes; let *P* ^+^and *P* ^−^ represent the set of forward arcs and backward arcs in the augmented route, respectively. Let *p*[*ij*] or *p*[*ijk*] represent the prior label of arc *ij* or arc *ijk* on augmented chain. Steps of the calculation are as follows.

**Step 1**

Give an initial feasible flow in the network. Zero flow can be treated as the initial feasible flow.

**Step 2**

Label the arc and find an augmented chain.

1. Label a random arc that regards the source node as the starting point with {*∞*, *h*_*ij*_}, while *h*_*ij*_ is the remaining possible maximum flow of the arc.
2. Pick the next arc linked to the prior labeled arc and then check it. (a) if the arc is the forward arc and the flow of the arc is less than the traffic capacity or (b) if the arc is backward arc and nonzero arc, then label it with {*p*[*ij*], *h*_*ij*_} or {*p*[*ijk*], *h*_*ijk*_}. *p*[*ij*] or *p*[*ijk*] is the prior labeled arc of the arc, and *h*_*ij*_ or *h*_*ijk*_ represents the remaining flow of the arc.
3. If a labeled arc's endpoint is the destination, it represents the augmented chain has been found. When continuous labeling cannot make the labeled arc point to the destination, it indicates that the augmented chain does not exist, so the calculation ends.

**Step 3**

Adjust the flow.

(1) Find the minimum value of arc *h*_*ij*_ or arc *h*_*ijk*_ on the augmented chain, and denote it with *h*.
(2) Adjust the flow according to the following formulas:
  (10) $$\begin{array}{c} f_{ij}=\{\begin{array}{cc} f_{ij} & \left(i,j\right)\in P\\ f_{ij}+h & \left(i,j\right)\in P^{+}\\ f_{ij}-h & \left(i,j\right)\in P^{-} \end{array} \end{array}$$

or

(11) $$\begin{array}{c} f_{ijk}=\{\begin{array}{cc} f_{ijk} & \left(i,j\right)\in P\\ f_{ijk}+h & \left(i,j\right)\in P^{+}\\ f_{ijk}-h & \left(i,j\right)\in P^{-} \end{array}. \end{array}$$

Then obtain the new possible flow *f*_1_.

**Step 4**

Cancel all labels and repeat Steps 2 and 3 constantly until new augmented chain cannot be found. At this moment, sets consisting of *f* of each arc are the maximum flow sets.

In fact, when maximum flows of each batch are implemented, we can know the time spent in each evacuation, as well as the evacuation time of each possible route. In the final evacuation, it is obvious that not all routes were able to evacuate in place, which means that evacuation routes consuming shorter time will be chosen preferentially. For example, before *m*th batch evacuation begins, it remains about time *q* after prior *m*-1 batches, and then the last evacuation batch only aim at the route with the evacuation time less than *q*, and *f*_*m*_ is the flow of final batch evacuation.

## 4. Evacuation Model Based on MC-MFM

Our evacuation model investigates a comparatively less clamant emergency situation, such as slight toxic gas leak. Everyone is required to be evacuated out of the danger zone. Here, people can rationally implement evacuation route planning and establish models with the goal of minimizing total evacuation time and maximizing the evacuation vehicle number. So evacuation problems in practical can be abstracted as a kind of minimum-cost maximum flow model (MC-MFM). Evacuations in multiple batches are still considered in this model, and only subsequent evacuation routes are implicit in the diagram. Specific model is as follows.

### 4.1. Premise and Hypothesis

In the network model with the object of obtaining the maximum flow within the given evacuation time, its basic premise and hypothesis are as follows.

1. Only one source node and one designated destination exist, and the number of evacuation vehicles are known.
2. Evacuation intervals of each batch are given and known.
3. Traffic capacity of each road section and each intersection are known.
4. The average traffic time on each road section and the average delay of different turnings of each intersection are known.
5. The goal is to minimize the total evacuation time and maximize evacuation vehicle number.

### 4.2. Symbol Definition

Nodes include source node, intersections, and evacuation destination. Similarly, arcs include road sections between the intersections, evacuation source node, and destination. Therefore, the network can be converted to network diagram *G* = (*V*, *L*, *D*, *C*, *T*, *U*).

 
*V* is the vertex, expressed by characters *i*, *j*, *k*.
 
*L* is the arc, while (*i*, *j*) is the arc from *i* to *j*.
 
*D* is a point weight set, while *d*_*ijk*_^*n*^ represents the delay of road section (*i*, *j*) to the road section (*j*, *k*) through *j* in *n*th batch.
 
*C* is also a point weight set, while *c*_*ijk*_ represents the traffic capacity of road section (*i*, *j*) through *j* to the road section (*j*, *k*).
 
*T* represents the arc weight set, while *t*_*ij*_^*n*^ represents average travel time of road section (*i*, *j*) in *n*th batch.
 
*U* represents the arc weight set, while *u*_*ij*_ represents the traffic capacity of road section (*i*, *j*).
 
*f* ^0^ is the total number of vehicles at source node.
 
*f*_*ij*_^*n*^ stands for the flow of arc (*i*, *j*) in *n*th batch.
 
*f*__*ijk*__^*n*^ represents the flow of arc (*i*, *j*) to arc (*j*, *k*) through *j* in *n*th batch.
 
*t* is the evacuation interval in each batch, also known as the valid time of traffic.

Besides, node  1 and node *m* stand for the source node and the designated destination, respectively.

### 4.3. Model Formulation

The above evacuation problem based on MC-MFM can be expressed as the following mathematical model:

(12)  Min⁡ z(f)=∑r=1∞tijrfijr+∑r=1∞dijkrfijkr, (i,j),(j,k)∈L

(13) s.t.0≤fijn≤uijn, (i,j)∈L

(14) $$\begin{array}{c} 0\leq f_{ijk}^{n}\leq u_{ijk}^{n},\;\left(i,j\right)\in L \end{array}$$

(15) $$\begin{array}{c} \overset{\infty }{\underset{r=1\;}{\sum }}\overset{n}{\underset{i=1}{\sum }}f_{1i}^{r}\;=\overset{\infty }{\underset{r=1\;}{\sum }}\overset{n}{\underset{i=1}{\sum }}f_{im}^{r}=f^{0} \end{array}$$

(16) fijn=∑k=1mfijkn   (i,j)∈L

(17) $$\begin{array}{c} f_{jk}^{n}\;=\overset{m}{\underset{i=1}{\sum }}f_{ijk}^{n}\;\left(j,k\right)\in L \end{array}$$

(18) fijn≥0, fijkn≥0 (i,j)∈L  , (j,k)∈L.

While formula (12) is the objective function; formulas (13) and (14) are the traffic capacity constraints of road sections and intersections, respectively, which means that the flow of road sections and intersections does not exceed the traffic capacity; formula (15) is the constraints of aggregate demand of evacuation, which illustrates that the total flow to the destination is equal to the total flow departing from source node; formula (16) refers to the flow of evacuation road section (*i*, *j*) in *n*th batch evacuation is equal to the total flow turning to other road sections at node *j*; formula (17) means that the flow of road sections (*j*, *k*) in the *n*th batch evacuation is equal to the total flow turning from other road sections to this road section through node *j*, and formula (18) is nonnegative constraints.

### 4.4. Solutions

Likewise, this model can also be converted to linear programming and solved by simplex method, but obviously it is easier to be solved by our MC-MFM.

If we find the first minimum cost flow *M* with the cost of *a*, then we assume that there are numerous other cost flows existing with costs of *a* + *t*, *a* + 2*t*, and so on. Each time a feasible flow with minimum cost is found, we assume that there are numerous virtual routes. And hereby we declare that traffic capacities of these virtual routes are recovered and have the same value as the first batch route. In the process of looking for smaller cost flows, if a smaller cost is not less than *a* + *t*, then we take flow *M* in the second batch as the minimum cost possible flow, and so on. Let *P* represents the augmented route or the minimum cost route in the process of solution. *P* ^+^ and *P* ^−^ are the set of forward arcs and backward arcs in the augmented route. *p*[*i*] is the prior node of node *i* in the augmented route or the minimum cost route. The steps of calculation are as follows.

**Step 1**

Give an initial feasible flow *f*_0_, which can be zero.

**Step 2**

Find a minimum cost road *p* from node  1 to node *m* in this network diagram.

**Step 3**

Augment the unit flow along route *p* and add flow *h* in augmented route *P* ^+^or intersection turnings. Otherwise, reduce flow *h* in augmented route *P* ^−^ or intersection turnings. Then a new possible flow *f* ^1^ is formed.

**Step 4**

After one round of augmentation, wipe off the arcs which have been saturated and then repeat Steps 2 and 3 and continue to augment until *f* ^0^ has been fully evacuated out. Then calculate the total time.

Specific calculation methods of this model will be displayed in the case analysis.

## 5. Extension to One-to-Many Evacuation

The models mentioned above are the evacuation from one source node to one designated destination. To make the study go further and deeper, the evacuation from single source node to many designated destinations will be considered in this part.

As for the evacuation from single source node to many designated destinations, the destination is no longer a single parameter as *m* but expressed by many parameters such as *m*_1_, *m*_2_,…, *m*_*q*_. We only need to change formula (15) to ∑*f*_1*i*_^*n*^ = ∑∑*f*_*im*_^*n*^, which represents the total flow arriving at multiple destinations is equal to the total flow of the source node.

We have two ways as follows to solve this problem.

The first one is to set a virtual evacuation destination *w* and then assume the delay time or travel time of all turning arcs and line arcs toward to arc (*m*_*i*_, *w*) are zero, and the traffic capacity is *∞*. In this way, evacuation from one source node to many designated destinations can be turned back to one source node to one designated destination.

The second one is to solve the model of one-to-many evacuation directly. In the process of calculation, the domain of the minimum cost route becomes broader. For example, if destinations *m*_1_, *m*_2_, and *m*_3_ exist, then we must find minimum cost routes from the source node to *m*_1_, *m*_2_, and *m*_3_, respectively and make comparisons to find the minimum cost route which will be regarded as the augmented route at this time. Though this method has differences with the first one, the essence is the same.

Certainly, emergency evacuation problem in one-to-many evacuation has a little difference from one-to-one evacuation. In other words, multiple evacuation destinations may have multiple capacity limitations, which require us to consider whether the evacuation flow toward certain destination will reach the maximum capacity.

## 6. Case Analysis

As we all know, Beijing is a big city with high concentration of population, especially in the downtown. Once some hazardous emergencies occur, it is likely to cause very serious losses. Under such circumstances, this paper will consider the specific route map within the second ring of Beijing, and the second ring road will be abstracted into network diagrams. Since the routes of the second ring road are comparatively complex, the abstracted route network diagrams are simplified according to importance, and more actual values for each parameter are given to ensure the accuracy of case analysis.

### 6.1. Case Description

#### 6.1.1. Selection of Network Diagrams

**Figure 1:**
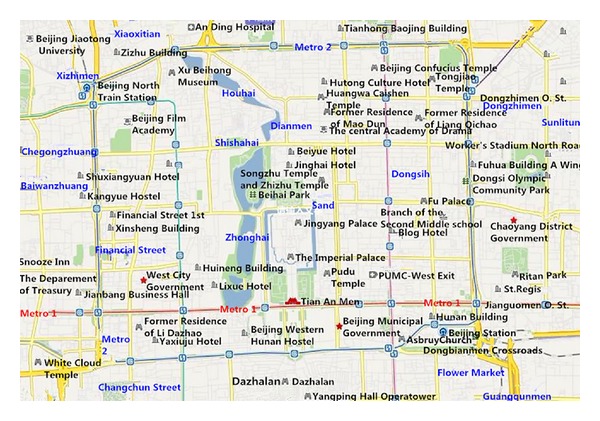
Beijing's second ring road.

This case analysis will calculate separately according to two different kinds of evacuation model, which is on the basis of actual second ring road map of Beijing, as shown in Figure 1.

**Figure 2:**
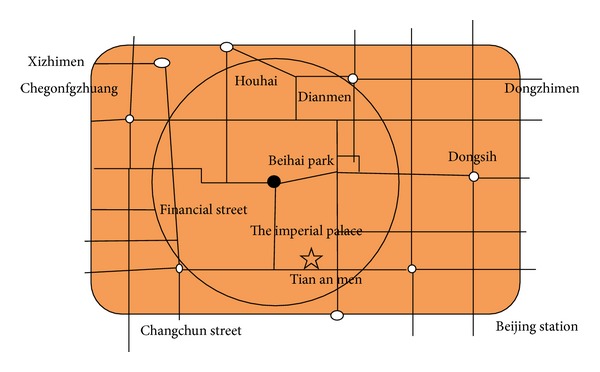
Route network of Beijing's second ring road.

According to Figure 1, we abstract its main routes to form a network route map, see Figure 2.

Assume that the black spot in Figure 2 is the source node. It is located between the imperial palace and the Beihai Park, which is also the area with the most intensive stream of people. Small white circles represent the assumed possible destinations. We assume that the big circle is the range of evacuation, and it is required that people should be evacuated to a destination or multiple destinations out of this circle.

**Figure 3:**
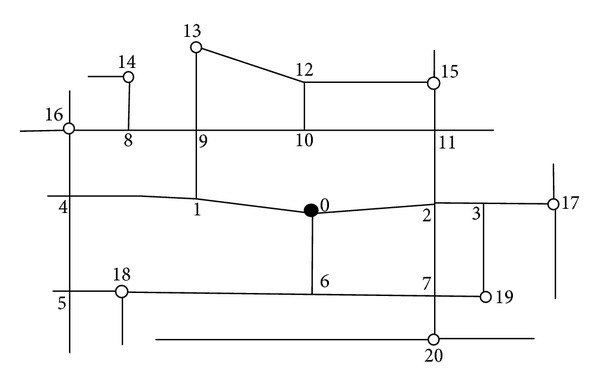
Simplified diagram of Beijing's second ring road traffic routes.

In order to calculate more conveniently, this paper ignores some minor roads and only considers main roads, so this road map will be simplified to Figure 3.

#### 6.1.2. Parameter Setting

Based on the simplified figure (Figure 3), we can conclude that there are eight T-crossroads, 1, 3, 4, 6, 8, 10, 11, and 12, and three crossroads, 2, 7, and 9.

According to the situation of actual evacuation, some road sections and intersection turnings should be regulated, and some possible traffic control measures should be implemented. Possible routes of each road section and accessible intersection turning will be described, and the traffic capacity and the amount of time consumed, including the travel time at road sections and the delay time at intersection turning, will be illustrated, see Tables 1 and 2.

Possible road sections and intersection turnings described in the above two tables are designed based on the traffic control measures. For example, there is no turning (3, 2, 11) at intersection 2 and turning (8, 9, 13) at intersection 9. In addition, we also assume that destinations will not be taken as the midpoint of other evacuation routes. For instance, there are no similar turnings as (19, 3, 17) and (15, 12, 13), and so forth. Of course, limitations of these control measures make calculations more convenient but do not affect the accuracy of the entire case.

### 6.2. Emergency Evacuation Based on MFM

#### 6.2.1. Problem Description

**Figure 4:**
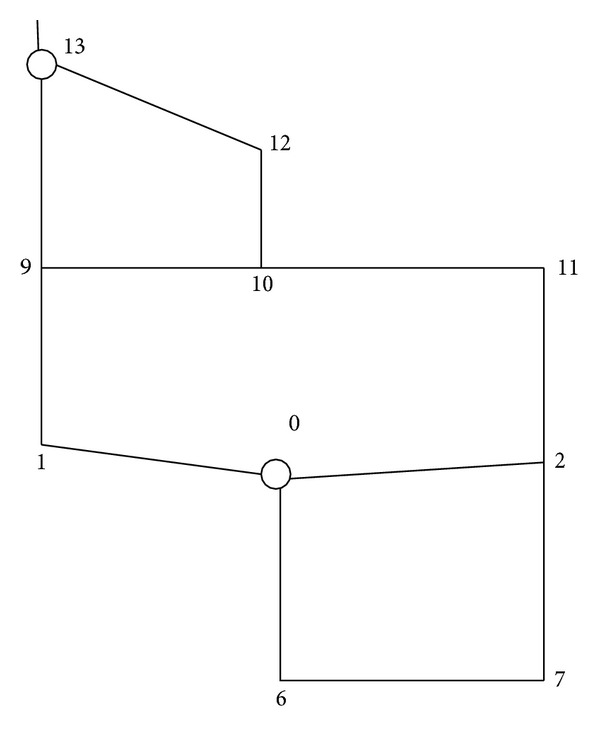
Simplified road sections in one-to-one evacuation.

Figure 3 is marked with multiple destinations, hereby we consider one-to-one evacuation at first and select node 13 as the destination. In order to calculate conveniently, we still assume that evacuation routes cannot pass other alternative destinations beside destination node 13. Thus, evacuation route network diagram can be simplified in Figure 4.

As the intersections 1, 6, 11, and 12 are no longer T-roads and intersections 2, 7, and 9 are also no longer crossroads, the delay time of each intersection turning will change inevitably. The traffic capacity and the amount of time consumed, including travel time and delay time of each road section and intersection turnings, are showed in Table 3.

In this paper, the total evacuation time is limited to 60 mins and the allowed evacuation time in each batch is 16 mins.

#### 6.2.2. Solutions

The first thing is to obtain the value of *m* and *m* = ⌈*T*/*t*⌉ = ⌈60/16⌉ = 4. The evacuation time in the fourth batch is only 12 mins.

The second thing is to get the static maximum flow *f*_1_.

**Step 1**

Let initial flow *f*_0_ be zero.

**Step 2**

Label arc (0, 1) with {*∞*, 20}, then label (0, 1, 9) with {(0,1), 16}; next, label (1, 9) with {(0,1, 9), 16} and label (1, 9, 13) with {(1,9), 8}}, then label (9, 13) with {(1,9, 13), 16}}, so the first round of labeling ends. Since *h*_1−9−13_ is the minimum among *h*_*ij*_ or *h*_*jk*_, adjust it with *h* = 8, and the flow of arc (0, 1), (0, 1, 9), (1, 9), (1, 9, 13), and (9, 13), namely, the flows on augmented chain *P*, is increased to 8. Arc (1, 9, 13) is saturated.

Label the arc with the next three rounds with adjustment.

**Step 3**

Since the limit time in the fourth batch is 12 mins. Therefore some routes, in which the travel time and the delay time are significantly more than 12 mins, will not be considered. Only route (0-1-9-13) is possible, and its flow is 8.

**Step 4**

Calculate *f*_*T*_ = *f*_1_ + *f*_2_ + *f*_3_ + *f*_4_ = 26. Specific evacuation routes are as shown in Table 4.

Due to the evacuation only towards a fixed destination, the delay time of some intersections is shorter than one-to-many evacuations. In our case, the traffic control measures have been considered. For example, road section (9, 10) is a two-way traffic, and most road sections are only one-way traffic.

### 6.3. Emergency Evacuation Based on MC-MFM

Then we continue to consider one-to-one evacuation with node 13 as the destination. For the simplicity of calculation, we still assume that the evacuation route cannot pass other alternate evacuation destinations except node 13. Thus, network diagram of evacuation routes is also illustrated in Figure 4. In addition, the total number of vehicles in the source node *f*_0_ is 60 hundred vehicles.

We need to find the minimum cost route and the augment route on this way until evacuation flow reaches the target value. The approach used here is almost like the Dijkstra algorithm, but the review for the routes with minimum cost has been changed into the review for arcs.

The first thing is to obtain the minimum cost route, see Table 5.

It can be seen from Table 5 that the minimum cost of route 0-1-9-13 is 10. As the bottleneck traffic flow on this route is arc (1, 9, 13), which is 8, this route is the augmented chain. When we increase its traffic flow about 8, arc (1, 9, 13) of the first batch is saturated at this moment, and the total route cost is 80, namely 8000 mins. In addition, subsequent batches of this route should be considered in the course of finding the minimum cost route.

As illustrated in Table 5, since the first batch route 0-2-11-10-12-13 does not include arc (1, 9, 13), the total cost of this route is 16, which is more than the virtual route's minimum value in the preamble, and thus the minimum cost route is route 0-1-9-13 in the second batch. As the bottleneck of traffic flow is arc (1, 9, 13), which is 8, the traffic capacity on this augmented chain is increased with 8, and arc (1, 9, 13) in the second batch is saturated at this moment with the total route cost of 112, namely 11200 mins.

We repeat the above steps according to Table 5 and obtain the following results in Table 6.

### 6.4. Case Analysis of One-to-Many Evacuation

Simple case calculations are implemented in the above section for the one-to-one evacuation problem on the basis of MC-MFM. In this section, the form of evacuation is extended to one-to-many evacuation. According to the second ring road map of Beijing, the evacuation destinations include eight nodes, which are nodes 13, 14, 15, 16, 17, 18, 19, and 20. In specific evacuation, the problems of route optimization are ensured based on MFM.

To keep the comparability with the case in the previous calculation, the total number of vehicles in the source node is still 60. The numerical values of specific capacity and the amount of time consumed are shown in Table 1. Source node is node 0, and evacuation destinations are 13, 14, 15, 16, 17, 18, 19, and 20.

In spite of being a different form, we still use label censorship method for calculation. Specific calculation process is shown in Table 7.

As illustrated in Table 7, we can find the minimum cost route 0-6-18 with the cost of 8. As the bottleneck of traffic capacity on arc (0, 6, 18) is 8, this route is augmented chain and traffic flow should be increased with 8. After that, arc (0, 6, 18) in the first batch is saturated, and the total route cost is 64. In addition, in the process of finding the minimum cost routes in the next step, subsequent batches should be considered.

Similarly, route 0-1-4-16 in the first batch does not include the arc (0, 6, 18), and its cost is 10, which is the minimum cost in the remaining viable routes, so this route is augmented chain. Since the bottleneck of traffic flow is arc (4, 16), which is 10, traffic flow on this augmented chain can be increased with 10. At this point, arc (4, 16) in the second batch is saturated, and the total route cost is 100.

We repeat the above steps according to Table 7 and obtain the following results, see Table 8.

Compared with one-to-one evacuation, one-to-many evaluation will take just 13 minutes, which is much faster and more efficient. Therefore, we draw conclusions as follows: under normal circumstances, when a certain emergency occurs and the emergency evacuation is required, multiple destinations for the evacuation are quicker and more efficient than only one destination. Therefore, in order to prevent heavy casualties caused by major emergencies and improve evacuation efficiency, more evacuation roads and shelters should be built in the places with high population density and high occurrence of emergencies.

## 7. Conclusions

Appropriate traffic control measures must be taken into account in evacuation route optimization. Overall, this paper explores evacuation optimization and planning of evacuation routes, considering some traffic control measures. We abstract the road network as directive network, taking the vehicles waiting for evacuation in the source node as flows and evacuation road sections along the road as arcs which are under traffic control, such as one-way, two-way, delay, and so forth. Besides, the traffic capacity of intersections is expressed as the weights of network diagram. After model formulation, we use graph theory method to solve our model and then verify its feasibility.

Based on the case analysis to the second ring of Beijing, we present evacuation optimization and planning after emergencies happen. Although we make a lot of prerequisites and assumptions for modeling and calculation, there are still some issues remained to be explored as follows.

1. Our paper considers the optimization of route under traffic control measures, but these measures like one-way, two-way, forbidding for passing, and intersection delay are finite. However, some measures with instant changes are not considered, and intersection delays are often changed in reality. All of the above factors are also required to be considered in future study.
2. We establish mathematical model and use graph theory, aiming at obtaining the maximum evacuation vehicle number with the minimum total time. In fact, we generally need to consider many factors in a variety of situations, and goals should not be single. In order to develop more realistic models to solve actual-world problems, we need to consider the integrated application of goal programming and graph theory when multiple goals should be achieved.

## Figures and Tables

**Table 1:** Possible road sections of emergency evacuation.

Starting node of road sections	Possible road sections	Traffic capacity (hundreds of vehicles)	Travel time (minutes)
0	(0, 1)	20	2
	(0, 2)	20	2.5
	(0, 6)	20	3

1	(1, 4)	16	2
	(1, 9)	16	2

2	(2, 3)	16	2
	(2, 7)	10	3
	(2, 11)	18	2

3	(3, 17)	10	1
	(3, 19)	10	3

4	(4, 5)	10	3
	(4, 16)	10	2

5	(5, 18)	8	2

6	(6, 7)	12	2.5
	(6, 18)	10	3

7	(7, 2)	10	3
	(7, 19)	16	2
	(7, 20)	12	1

8	(8, 14)	8	1
	(8, 16)	8	0.8

9	(9, 8)	12	1
	(9, 10)	12	3
	(9, 13)	16	3

10	(10, 9)	12	3
	(10, 11)	12	1.5
	(10, 12)	16	1

11	(11, 10)	12	1.5
	(11, 15)	16	1

12	(12, 13)	10	4
	(12, 15)	10	1.5

**Table 2:** Possible turnings of emergency evacuation.

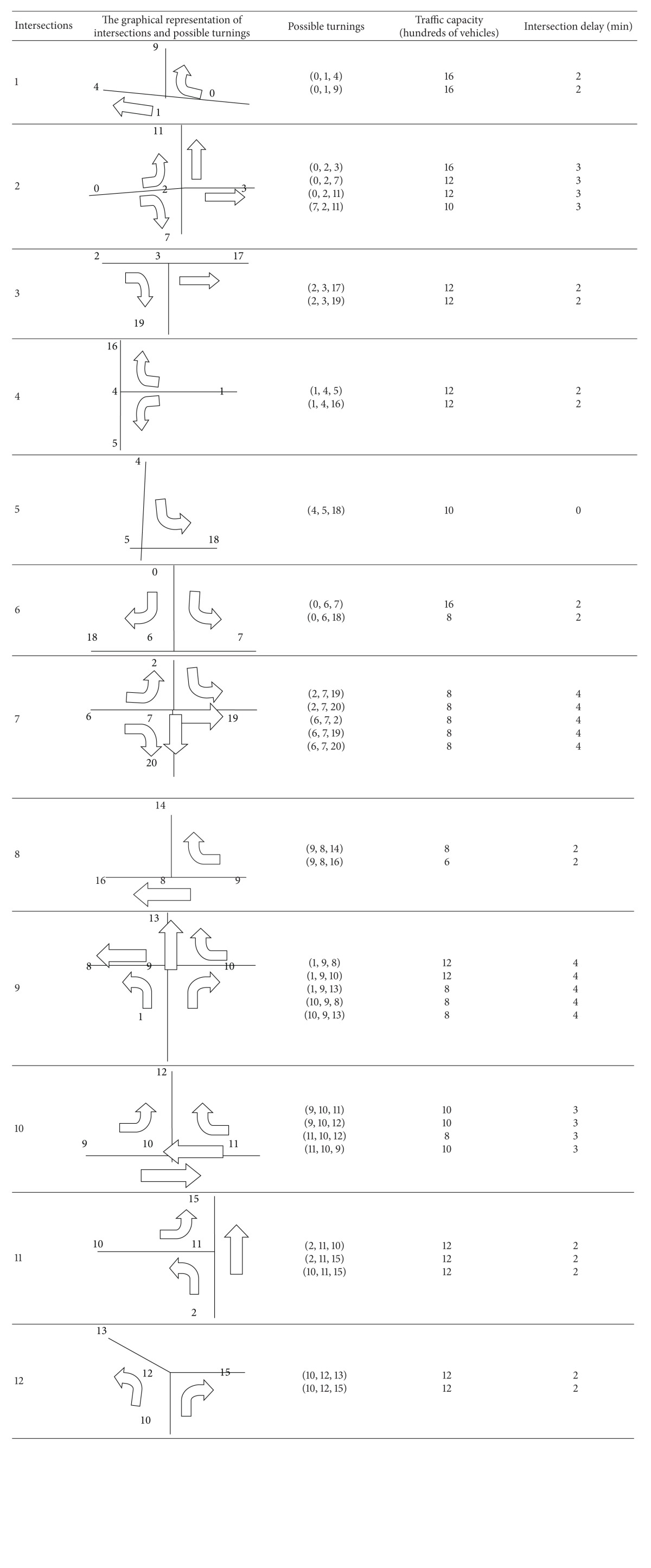

**Table 3:** Traffic capacity and time schedule of each road section.

Arc	Traffic capacity (hundreds of vehicles)	Travel time or delay (min)	Arc	Traffic capacity (hundreds of vehicles)	Travel time or delay (min)
(0, 1)	20	2	(0, 1, 9)	16	0
(0, 2)	20	2.5	(0, 2, 11)	12	2
(0, 6)	20	3	(0, 6, 7)	16	0
(1, 9)	16	2	(1, 9, 10)	12	3
(2, 11)	18	2	(1, 9, 13)	8	3
(6, 7)	12	2.5	(2, 11, 10)	12	0
(7, 2)	10	3	(6, 7, 2)	8	0
(9, 10)	12	3	(7, 2, 11)	10	2
(9, 13)	16	3	(9, 10, 12)	10	3
(10, 9)	12	3	(10, 9, 13)	8	3
(10, 12)	16	1	(10, 12, 13)	12	0
(11, 10)	12	1.5	(11, 10, 9)	10	3
(12, 13)	10	4	(11, 10, 12)	8	3

**Table 4:** Optimal routes of one-to-one evacuation on MFM.

Round	Route	Flow
*f*_1_ *f*_1_	0-1-9-10-12-13	8
*f*_2_	0-2-11-10-12-13	2
*f*_3_	0-2-11-10-9-13	8
*f*_4_	0-1-9-13	8
*f*_*T*_	4	26

**Table 5:** Minimum-cost emergency evacuation route.

Minimum cost from the source node	2	2	2.5	3	3
Reviewed arc	(0, 1)	(0, 1, 9)	(0, 2)	(0, 6)	(0, 6, 7)
The cost of associated arc segments departing from the source node	(0, 1, 9), 2	(1, 9), 4 (0, 1, 9), 7	(0, 2, 11), 4.5	(0, 6, 7), 3	(6, 7), 5.5

Minimum cost from the source node	4	4.5	5.5	5.5	6.5
Reviewed arc	(1, 9)	(0, 2, 11)	(6, 7)	(6, 7, 2)	(2, 11)
The cost of associated arc segments departing from the source node	(1, 9, 10), 7	(2, 11), 6.5	(6, 7, 2), 5.5	(7, 2), 8.5	(2, 11, 10), 6.5

Minimum cost from the source node	6.5	7	7	8	8.5
Reviewed arc	(2, 11, 10)	(1, 9, 10)	(1, 9, 13)	(11, 10)	(7, 2)
The cost of associated arc segments departing from the source node	(11, 10), 8	(9, 10), 10	(9, 13), 10	(11, 10, 9), 11 (11, 10, 12), 11	(7, 2, 11), 10.5

Minimum cost from the source node	10	10	10	10.5	11
Reviewed arc	(9, 10)	(9, 13)	(9, 10)	(7, 2, 11)	(11, 10, 12)
The cost of associated arc segments departing from the source node	(9, 10, 12), 13	Reach the designated destination	(9, 10, 12), 13	(2, 11), 12.5	(10, 12), 12

Minimum cost from the source node	11	12	12	12.5	12.5
Reviewed arc	(11, 10, 9)	(10, 12)	(10, 12, 13)	(2, 11)	(2, 11, 10)
The cost of associated arc segments departing from the source node	(10, 9), 14	(10, 12, 13), 12	(12, 13), 16	(2, 11, 10), 12.5	(11, 10), 14

Minimum cost from the source node	13	14	14	14	14
Reviewed arc	(9, 10, 12)	(10, 12)	(10, 12, 13)	(10, 9)	(11, 10)
The cost of associated arc segments departing from the source node	(10, 12), 14	(10, 12, 13), 14	(12, 13), 18	(10, 9, 13), 17	(11, 10, 12), 17 (11, 10, 9), 17

Minimum cost from the source node	16	17	17	17	18
Reviewed arc	(12, 13)	(10, 9, 13)	(11, 10, 9)	(11, 10, 12)	(10, 12)
The cost of associated arc segments departing from the source node	Reach the designated destination	(9, 13), 20	(10, 9), 20	(10, 12), 18	(10, 12, 13), 18

Minimum cost from the source node	18	18	20	20	22
Reviewed arc	(10, 12, 13)	(12, 13)	(9, 13)	(10, 9)	(12, 13)
The cost of associated arc segments departing from the source node	(12, 13), 22	Reach the designated destination	Reach the designated destination	(10, 9, 13), 23	Reach the designated destination

Minimum cost from the source node	23	26			
Reviewed arc	(10, 9, 13)	(9, 13)			
The cost of associated arc segments departing from the source node	(9, 13), 26	Reach the designated destination			

**Table 6:** Optimal routes of one-to-one evacuation on MC-MFM.

Serial number	Route	Number of batch	Flow	Total time (minutes)
*f*_1_	0-1-9-13	4	32	22
*f*_2_	0-2-11-10-12-13	2	16	
*f*_3_	0-1-9-10-12-13	2	8	
*f*_4_	0-2-11-10-9-13	1	4	
*f*_*T*_	4	9	60	

**Table 7:** Optimal routes of one-to-many emergency on MC-MFM.

Minimum cost from the source node	2	2.5	3	4	4
Reviewed arc	(0, 1)	(0, 2)	(0, 6)	(0, 1, 4)	(0, 1, 9)
The cost of associated arc segments departing from the source node	(0, 1, 4), 4 (0, 1, 9), 4	(0, 2, 3), 5.5	(0, 6, 7), 5 (0, 6, 18), 5	(1, 4), 6	(1, 9), 6

Minimum cost from the source node	5	5	5.5	5.5	5.5
Reviewed arc	(0, 6, 7)	(0, 6, 18)	(0, 2, 11)	(0, 2, 7)	(0, 2, 3)
The cost of associated arc segments departing from the source node	(6, 7), 7.5	(6, 18), 8	(2, 11), 7.5	(2, 7), 8.5	(2, 3), 7.5

Minimum cost from the source node	6	6	7.5	7.5	7.5
Reviewed arc	(1, 4)	(1, 9)	(2, 3)	(2, 11)	(6, 7)
The cost of associated arc segments departing from the source node	(1, 4, 5), 8 (1, 4, 16), 8	(1, 9, 8), 10 (1, 9, 10), 10 (1, 9, 13), 10	(2, 3, 17), 9.5 (2, 3, 19), 9.5	(2, 11, 10), 9.5 (2, 11, 15), 9.5	(6, 7, 2), 11.5 (6, 7, 19), 11.5 (6, 7, 20), 11.5

Minimum cost from the source node	8	8	8	8.5	9.5
Reviewed arc	(6, 18)	(1, 4, 5)	(1, 4, 16)	(2, 7)	(2, 3, 17)
The cost of associated arc segments departing from the source node	Reach the destination	(4, 5), 11	(4, 16), 10	(2, 7, 19), 12.5 (2, 7, 20), 12.5	(3, 17), 10.5

Minimum cost from the source node	9.5	9.5	9.5	10	10
Reviewed arc	(2, 3, 19)	(2, 11, 10)	(2, 11, 15)	(1, 9, 8)	(1, 9, 13)
The cost of associated arc segments departing from the source node	(2, 19), 12.5	(11, 10), 11	(11, 15), 10.5	(9, 8), 11	(9, 13), 13

Minimum cost from the source node	10	10.5	10.5	11	11
Reviewed arc	(4, 16)	(3, 17)	(11, 15)	(4, 5)	(4, 5, 18)
The cost of associated arc segments departing from the source node	Reach the destination	Reach the destination	Reach the destination	(4, 5, 18), 11	(5, 18), 13

Minimum cost from the source node	11	11	11.5	11.5	11.5
Reviewed arc	(11, 10)	(9, 8)	(6, 7, 19)	(6, 7, 2)	(6, 7, 20)
The cost of associated arc segments departing from the source node	(11, 10, 9), 14 (10, 10, 12), 14	(9, 8, 14), 13 (9, 8, 16), 13	(7, 19), 13.5	(7, 2), 14.5	(7, 20), 12.5

Minimum cost from the source node	12.5	12.5	12.5	12.5	13
Reviewed arc	(3, 19)	(2, 7, 19)	(2, 7, 20)	(7, 20)	(9, 13)
The cost of associated arc segments departing from the source node	Reach the destination	(7, 19), 14.5	(7, 20), 13.5	Reach the destination	Reach the destination

**Table 8:** Optimal routes of one-to-many evacuations on MC-MFM.

Serial number	Route	Number of batch	Flow	Total time (minutes)
*f*_1_	0-6-18	2	16	13
*f*_2_	0-1-4-16	1	10	
*f*_3_	0-2-3-17	1	10	
*f*_4_	0-2-11-15	1		
*f*_5_	0-6-7-20	1	8	
*f*_6_	0-1-9-13	1	6	
*f*_*T*_	5	7	60	
